# Supplementary material for: Simultaneous Effects of Carboxyl Group-Containing Hyperbranched Polymers on Glass Fiber-Reinforced Polyamide 6/Hollow Glass Microsphere Syntactic Foams
Source: Polymers (Basel). 2022 May 7;14(9):1915. doi: 10.3390/polym14091915 (PMC9099570; doi:10.3390/polym14091915)
Supplement: Supplementary file 1 [file polymers-14-01915-s001.zip › polymers-1658513-supplementary.pdf]

# Supplementary Materials

## Simultaneous effect of carboxyl group-containing hyperbranched polymer on the glass fiber-reinforced polyamide 6/hollow glass microsphere syntactic foams

Jincheol Kim[1,†], Jaewon Lee[1,†], Sosan Hwang[1], Kyungjun Park[1], Sanghyun Hong[2], Seojin Lee[2], Sang Eun Shim[1,*], and Yingjie Qian[1,*]

[1] Department of Chemistry and Chemical Engineering, Education and Research Center for Smart Energy and Materials, Inha University, Incheon 22212, Republic of Korea

[2] H&A R&D Center, LG Electronics., LTD, 51 Gasan digital 1-ro, Geumcheon-gu, Seoul, 153802, Korea

[†] These authors contributed equally to this work.

[*]*Corresponding author. E-mail address*: seshim@inha.ac.kr for SE Shim, yjqian@inha.ac.kr for Y Qian

**Table S1.** DSC data for syntactic foams and a glass fiber reinforced PA 6 composite under non-isothermal crystallization.

| Sample code | $T_m$ (°C) | $\Delta H_f$ (J/g) | $X_c$ (%) | $T_c$ (°C) | $T_{onset}$ (°C) | $\Delta H_c$ (J/g) | $\Delta H_c^*$ (J/g) |
|---|---|---|---|---|---|---|---|
| PA 6 | 221.3 | 47.95 | 19.98 | 192.5 | 196.3 | 55.46 | 55.46 |
| PA 6/HGM 5 | 221.5 | 45.92 | 20.36 | 191.7 | 195.8 | 51.11 | 54.39 |
| PA 6/HGM 20 | 222.1 | 41.94 | 21.63 | 190.3 | 195.0 | 46.24 | 57.23 |
| PA 6/GF 5 | 221.4 | 47.77 | 21.23 | 192.3 | 196.2 | 54.25 | 57.86 |

**Table S2.** Crystallization kinetic analysis of non-isothermal crystallization data for syntactic foams and a glass fiber reinforced composite.

| Sample code | n | $Z_t$ | $Z_c$ | $t_{1/2}$ (min) | Adj. R-Square |
|---|---|---|---|---|---|
| PA 6 | 3.5 | 1.981 | 1.071 | 0.74 | 0.9996 |
| PA 6/HGM 5 | 3.4 | 1.904 | 1.066 | 0.74 | 0.9998 |
| PA 6/HGM 20 | 3.4 | 1.365 | 1.032 | 0.82 | 0.9999 |
| PA 6/GF 5 | 3.6 | 1.871 | 1.065 | 0.76 | 0.9997 |

**Table S3.** Comparison of the mechanical properties of neat PA 6, syntactic foams, and a glass fiber reinforced PA 6 composite.

| Sample code | Specific gravity | Tensile strength (MPa) | Elongation (%) | Flexural modulus (MPa) | Flexural strength (MPa) |
|---|---|---|---|---|---|
| PA 6 | 1.125 | 80.2 ± 2.6 | 13.6 ± 13.8 | 2843 ± 73 | 35.3 ± 0.9 |
| PA 6/HGM 5 | 1.063 | 67.8 ± 1.3 | 8.4 ± 6.1 | 3023 ± 68 | 37.5 ± 0.8 |
| PA 6/HGM 10 | 1.018 | 62.9 ± 1.2 | 3.3 ± 0.8 | 3215 ± 28 | 39.9 ± 0.3 |
| PA 6/HGM 15 | 0.983 | 52.8 ± 1.5 | 2.1 ± 1.8 | 3360 ± 23 | 41.7 ± 0.3 |
| PA 6/HGM 20 | 0.940 | 47.6 ± 2.3 | 1.6 ± 0.4 | 3343 ± 46 | 41.5 ± 0.6 |
| PA 6/GF 5 | 1.167 | 90.4 ± 4.1 | 3.2 ± 1.6 | 3371 ± 74 | 41.8 ± 0.9 |

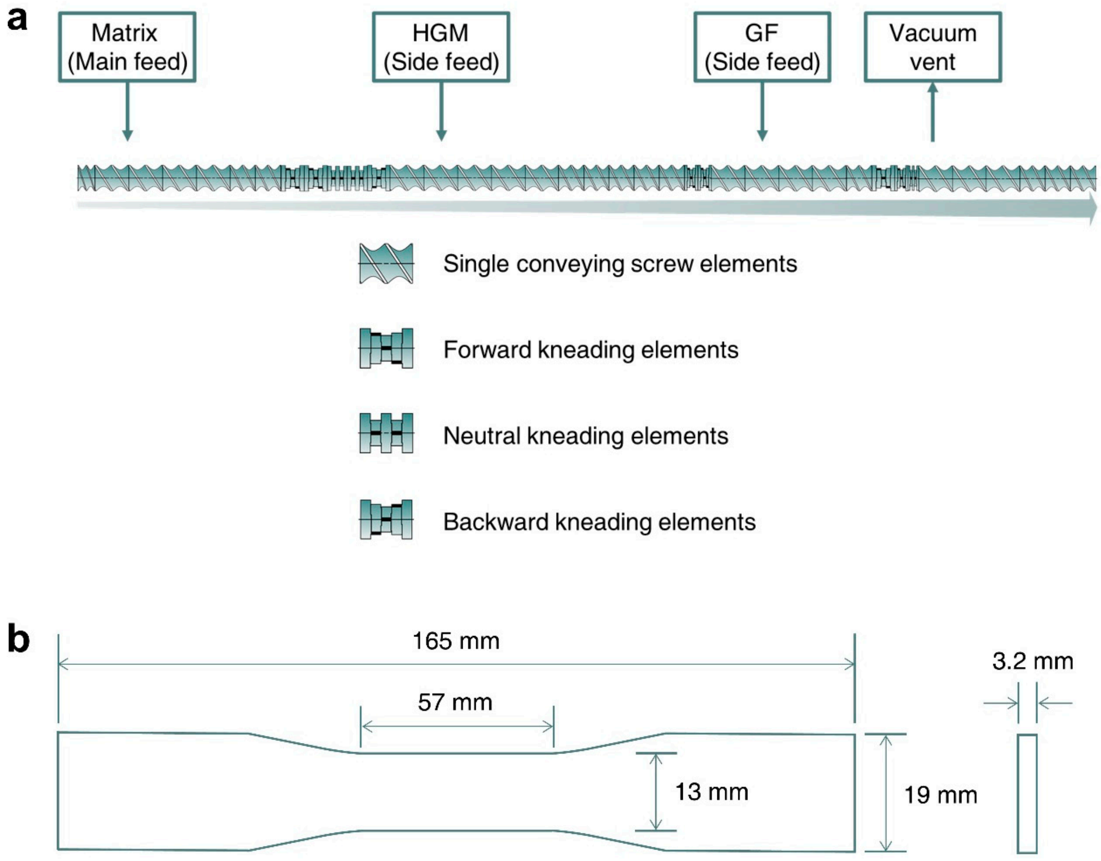


**Figure S1.** (a) Twin-screw configuration representing extrusion process starting from left to right. HGMs and GFs are side-fed in the middle. (b) Tensile test specimen dimension in accordance with ASTM D638 Type I standard. Gauge length of the specimen is 50 mm

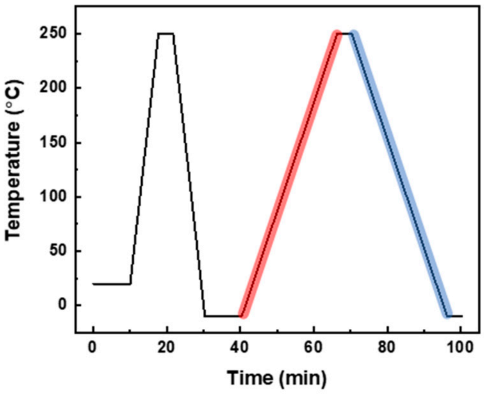


| | |
|---|---|
| #SEG. 1 | Isothermal at 20℃ for 10 min |
| #SEG. 2 | Heating at 30 ℃/min from 20 ℃ to 250 ℃ |
| #SEG. 3 | Isothermal at 250℃ for 4 min |
| #SEG. 4 | Cooling at 30 ℃/min from 250 ℃ to -10 ℃ |
| #SEG. 5 | Isothermal at -10℃ for 10 min |
| #SEG. 6 | Heating at 10 ℃/min from -10 ℃ to 250 ℃ |
| #SEG. 7 | Isothermal at 250℃ for 4 min |
| #SEG. 8 | Cooling at 10 ℃/min from 250 ℃ to -10 ℃ |
| #SEG. 9 | Isothermal at -10℃ for 4 min |

**Figure S2.** DSC profile to figure out the effect of HBP contents within RSF samples on non-isothermal crystallization behavior. Data was collected through Segment 6 on heating cycle to measure and calculate $T_m$, $\Delta H_f$, and $X_c$. Segment 8 was implemented on cooling cycle to measure and calculate $T_c$, $T_{onset}$, $\Delta H_c$, and $\Delta H_c^*$.

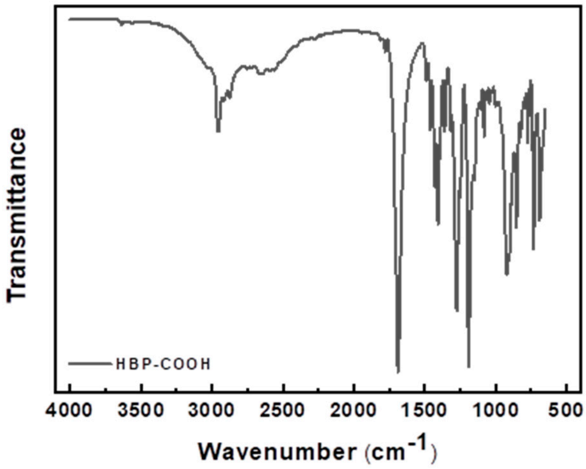


**Figure S3.** FT-IR spectra of HBP. OH stretching in the carboxyl group is 2952 $cm^{-1}$ and aliphatic O-H bending is 1427$cm^{-1}$. Aliphatic ester group of C-O stretching is 1191 $cm^{-1}$ and aliphatic ether group of C-O stretching is 1149 $cm^{-1}$.

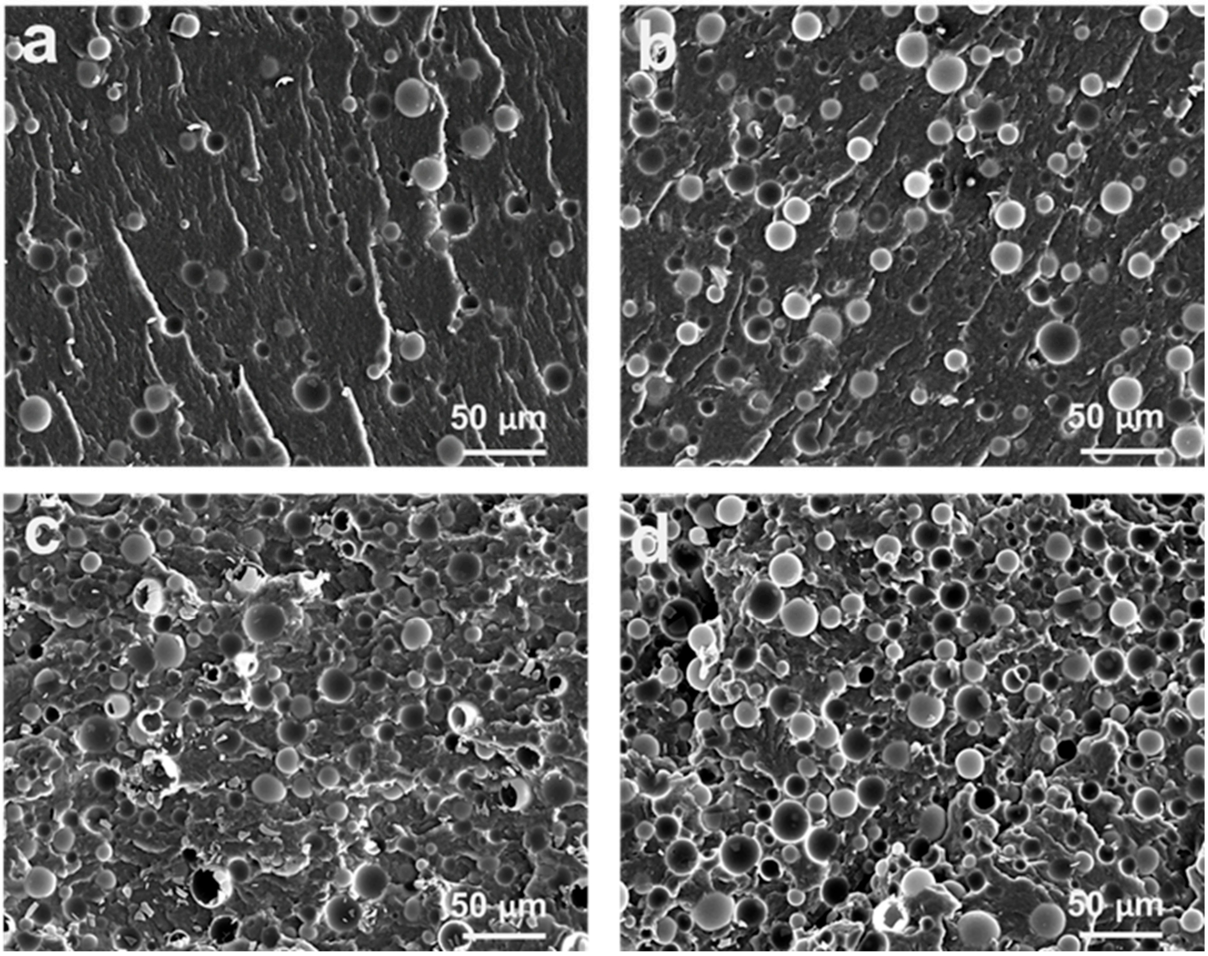


**Figure S4.** SEM microphotographs of syntactic foams on fractured surfaces after tensile test: PA 6/HGM 5 (a), PA 6/HGM 10 (b), PA 6/HGM 15 (c), and PA 6/HGM 20 (d).

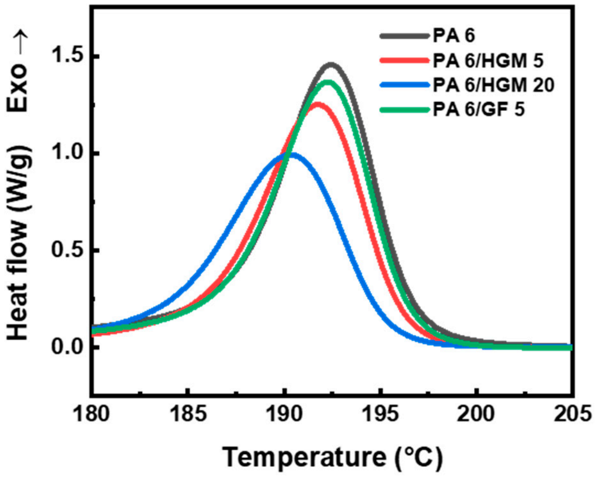


**Figure S5.** DSC thermograms of syntactic foams. The more the HGM was introduced, the lower the peak of crystallization temperature was found.

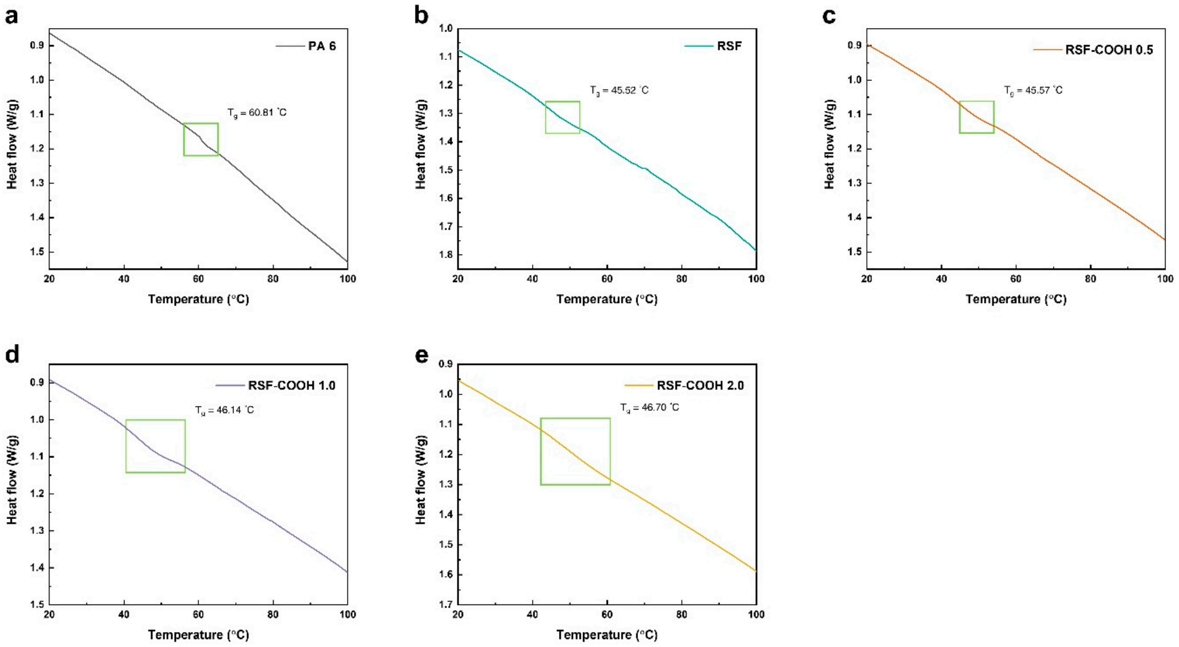


**Figure S6.** DSC thermograms ranging from 20 °C to 100 °C of the PA 6 (a), RSF (b), RSF-COOH 0.5 (c), RSF-COOH 1.0 (d), and RSF-COOH 2.0 (e) for the glass transition temperature (Tg) evaluation. Tg of each sample is depicted in the plots**.**

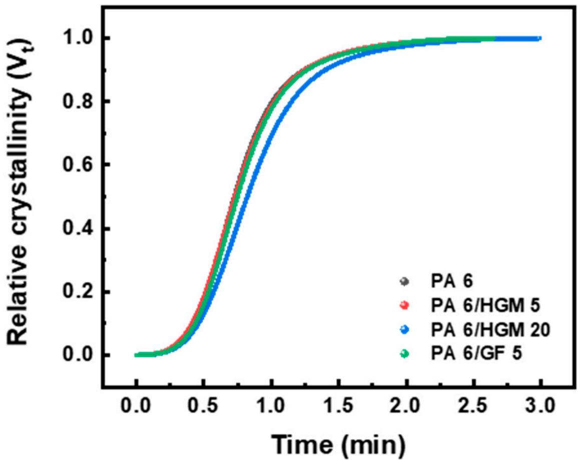


**Figure S7.** Plots of relative crystallinity ($V_t$) vs time for the non-isothermal crystallization of neat PA 6, syntactic foams with different HGM contents, and reinforced PA 6 composites.

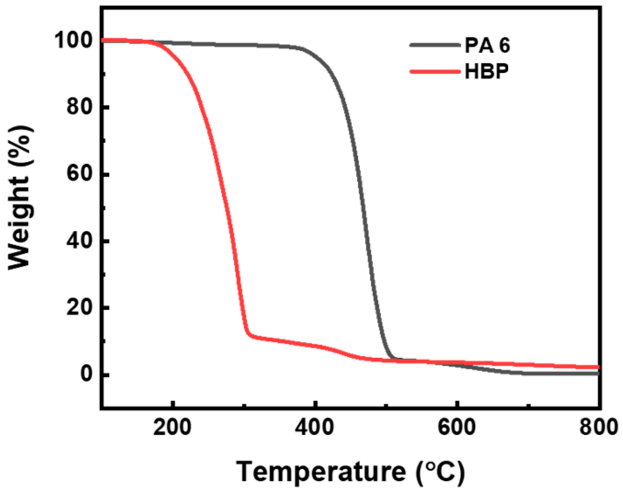

**Figure S8.** TGA thermograms of PA 6 and HBP.

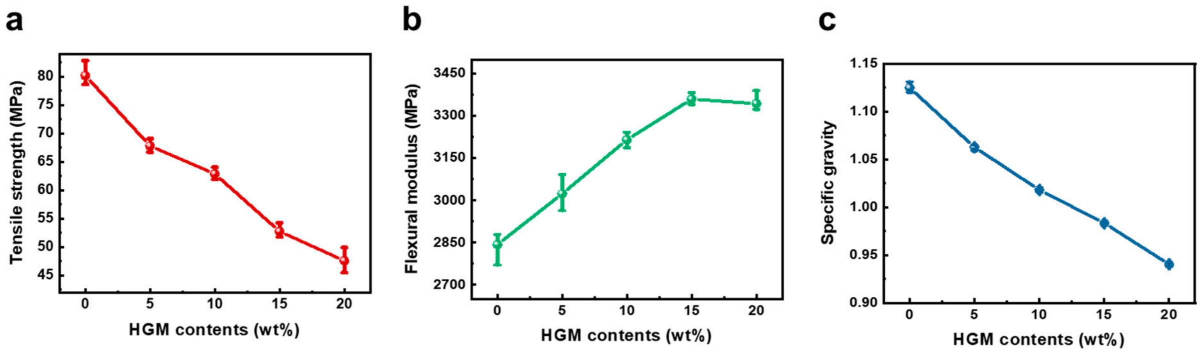


**Figure S9.** Tensile strength (a), flexural modulus (b), and specific gravity (c) of syntactic foams with various HGM contents.

$$\rho_{Inorganic\ ashes} = \frac{1}{\frac{w_{GF}}{\rho_{GF}} + \frac{W_{crushed\ HGM}}{\rho_{crushed\ HGM}}}$$

$$\%\ vol\ of\ HGM\ broken\ in\ process\ = \left[\frac{\left(\frac{1}{\rho_{HGM-input}}\right) - \left(\frac{1}{\rho_{crushed\ HGM}}\right)}{\left(\frac{1}{\rho_{HGM-input}}\right) - \left(\frac{1}{\rho_{HGM-100\%\ breakage}}\right)}\right]$$

**Figure S10.** Equations for calculating HGM breakage. The density of HGMs reaches 2.54 g/cm$^3$ at 100% breakage. By exposing the pellets to 550 °C for 2 hrs to remove the matrix (PA 6), true densities of the residual inorganic ash consisting of HGMs and GFs were measured by gas pycnometer, which were 0.4365 and 2.4510 g/cm$^3$, respectively.

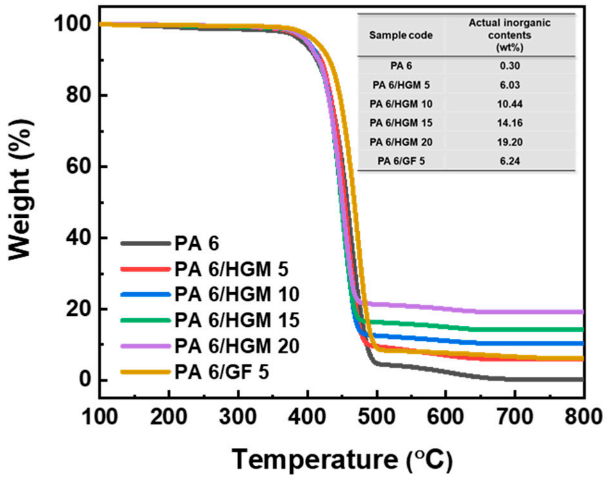


**Figure S11.** TGA thermograms of syntactic foams and a glass fiber reinforced PA 6 composite. Inset: actual inorganic filler contents.
